# Supplementary figures and images for: Functional stasis and changing habitat preferences among mammalian communities from the PETM of the Bighorn Basin, Wyoming
Source: Camb Prism Extinct. 2024 Dec 5;2:e20. doi: 10.1017/ext.2024.25 (PMC11895753; doi:10.1017/ext.2024.25)

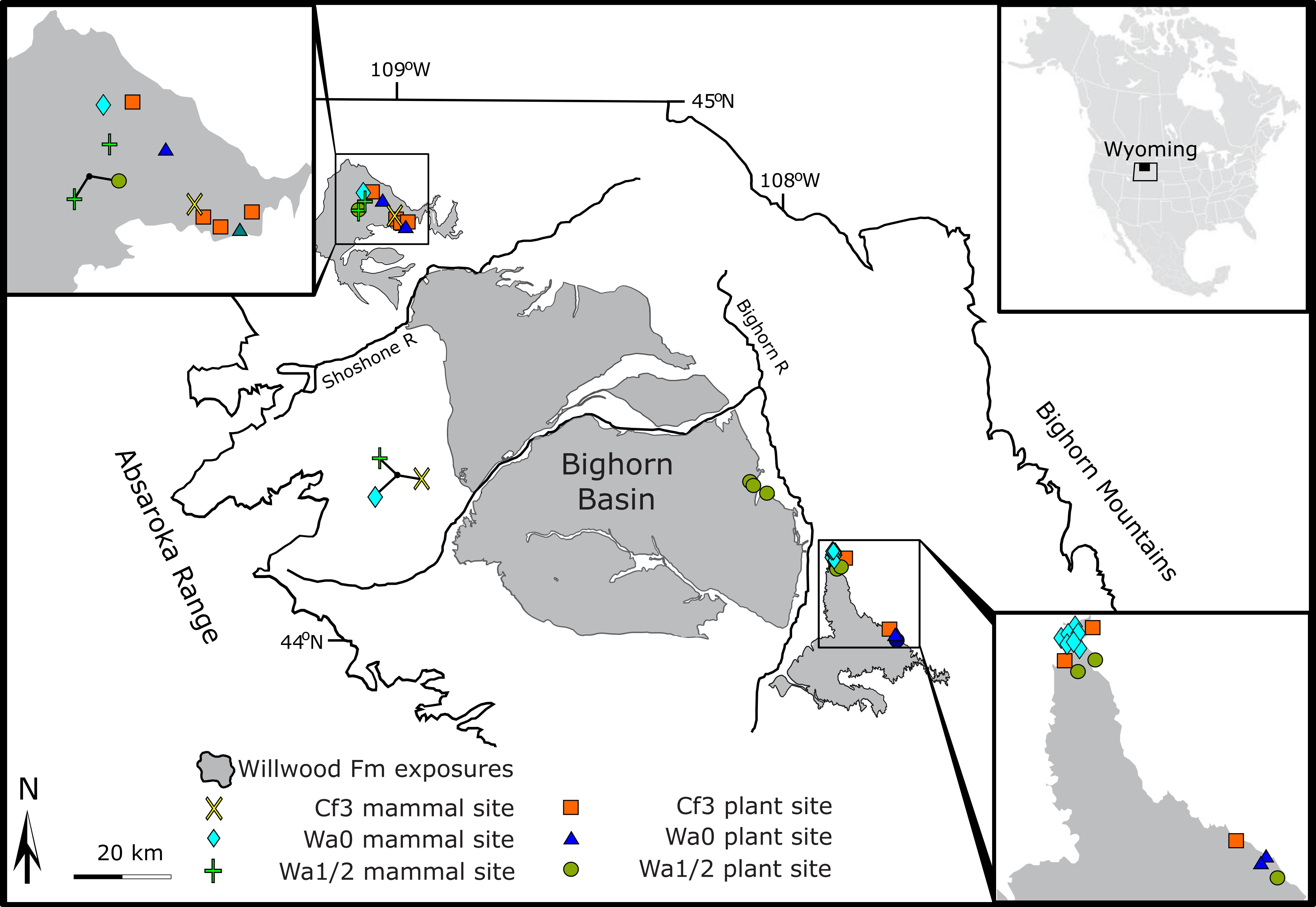

Supplement: Whittingham et al. supplementary material [file S2755095824000251sup001.zip › S2755095824000251sup001/fig1.jpg]

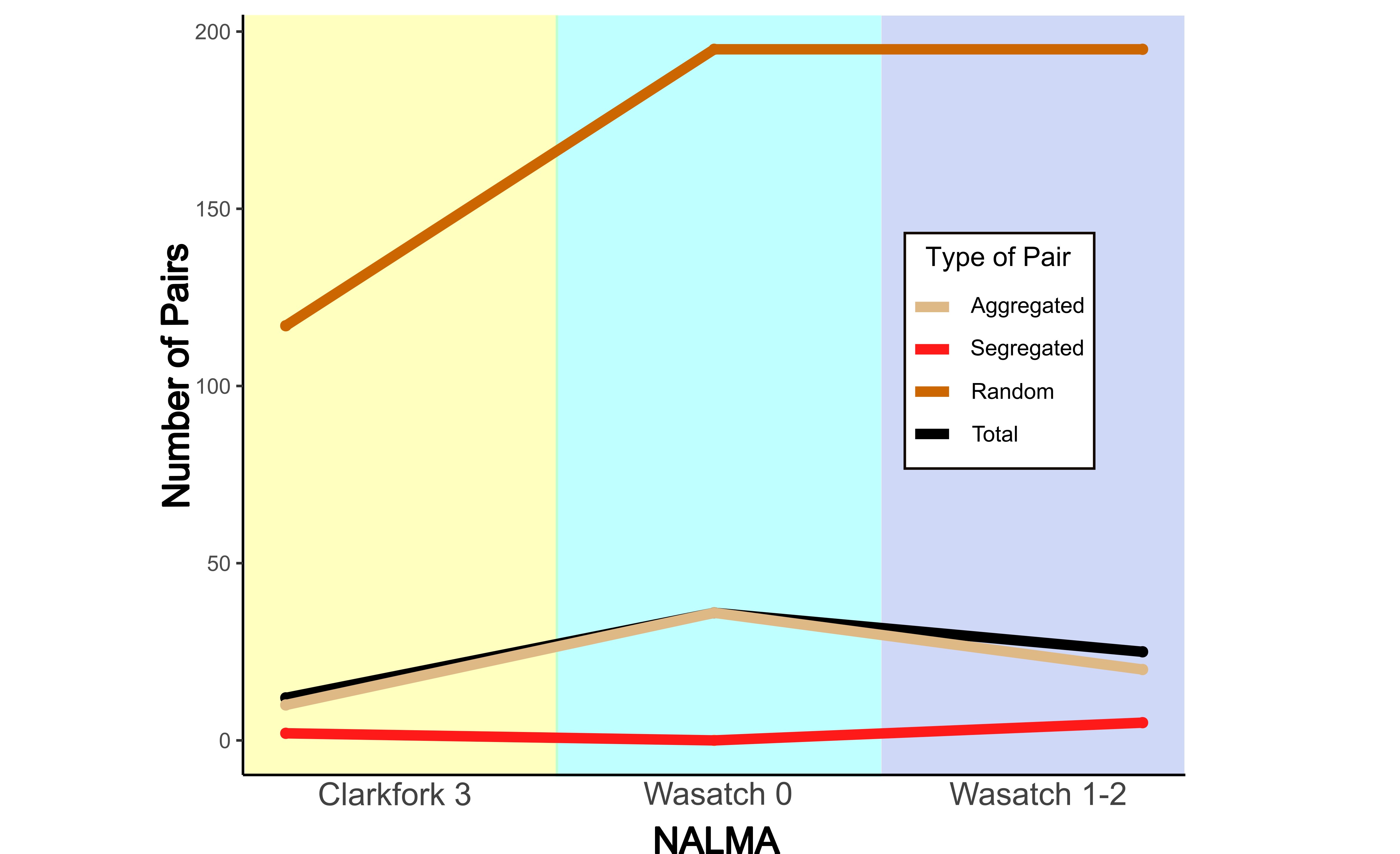

Supplement: Whittingham et al. supplementary material [file S2755095824000251sup001.zip › S2755095824000251sup001/Fig2.jpg]

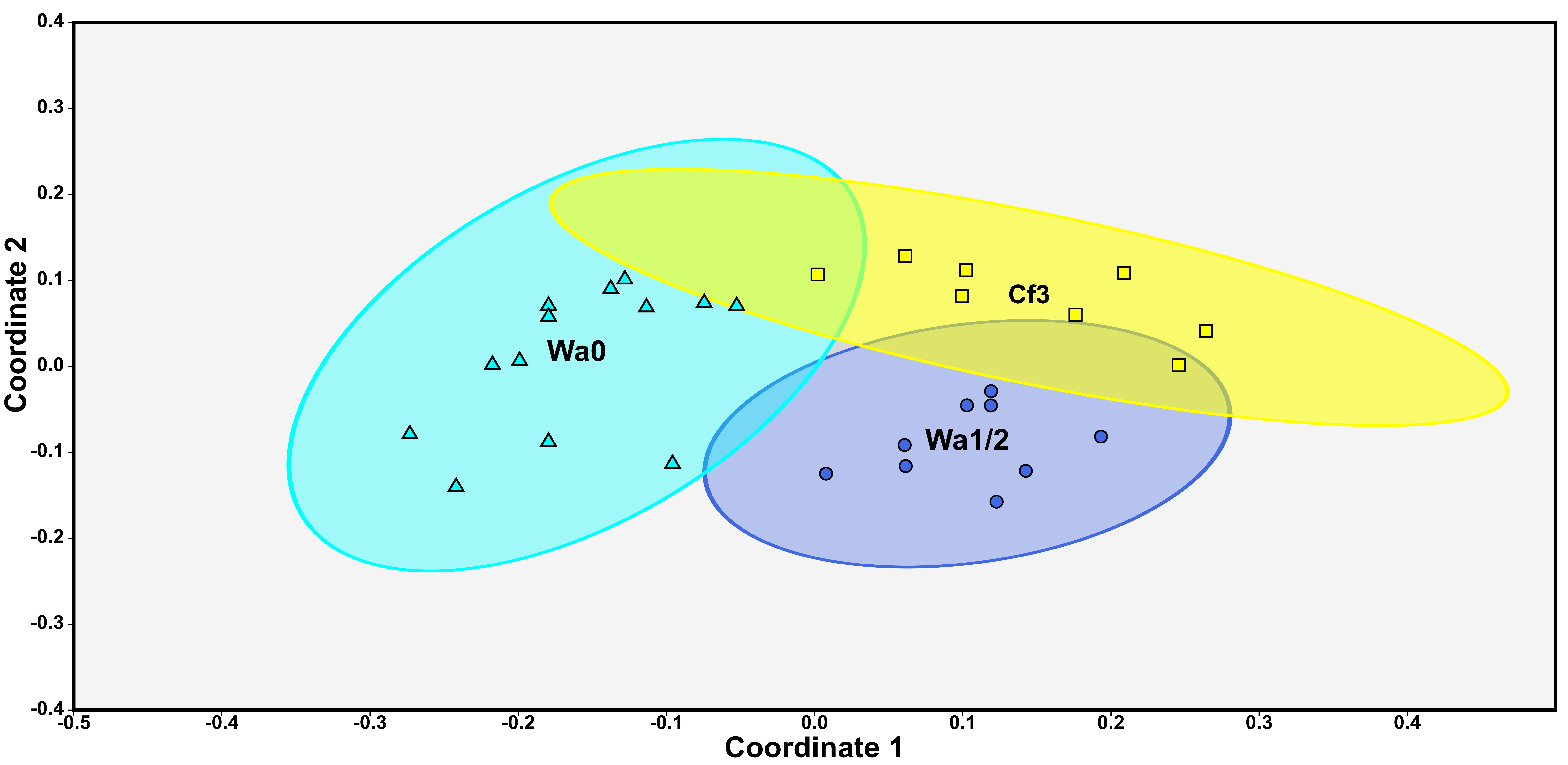

Supplement: Whittingham et al. supplementary material [file S2755095824000251sup001.zip › S2755095824000251sup001/fig3.jpg]

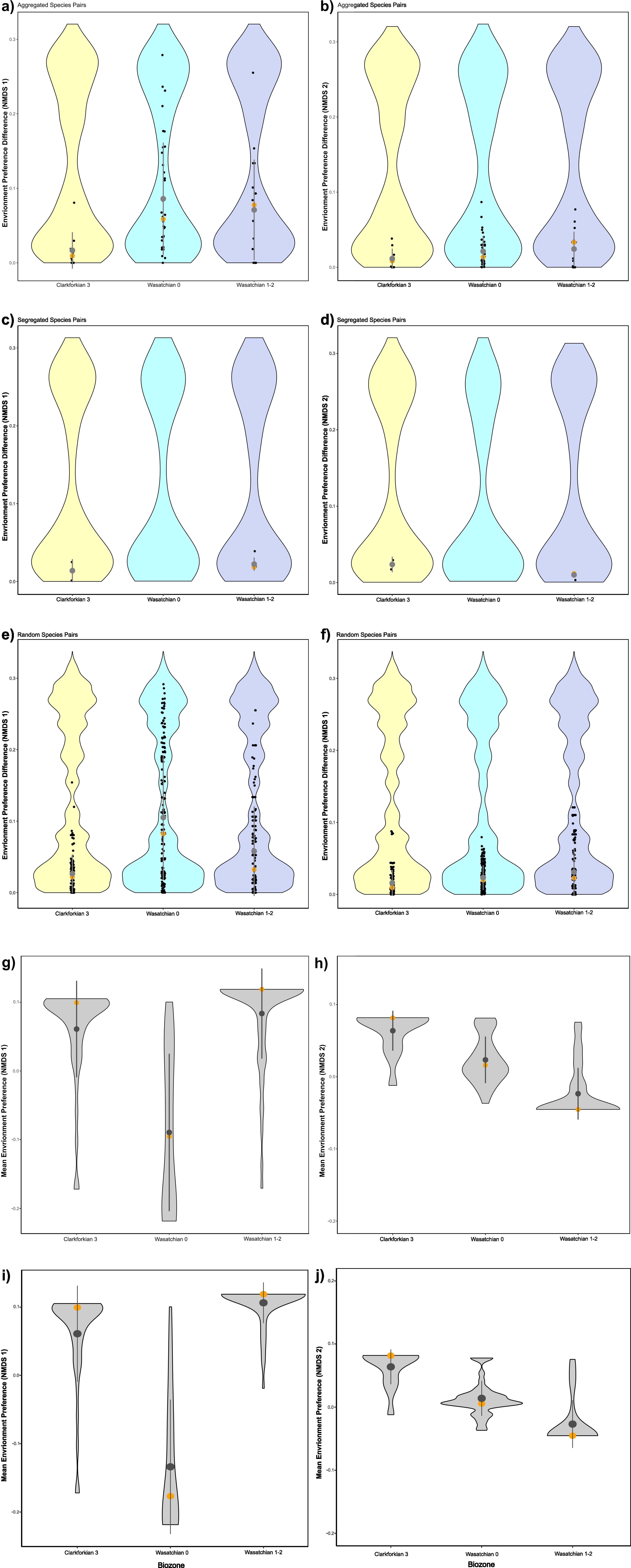

Supplement: Whittingham et al. supplementary material [file S2755095824000251sup001.zip › S2755095824000251sup001/fig4.jpg]

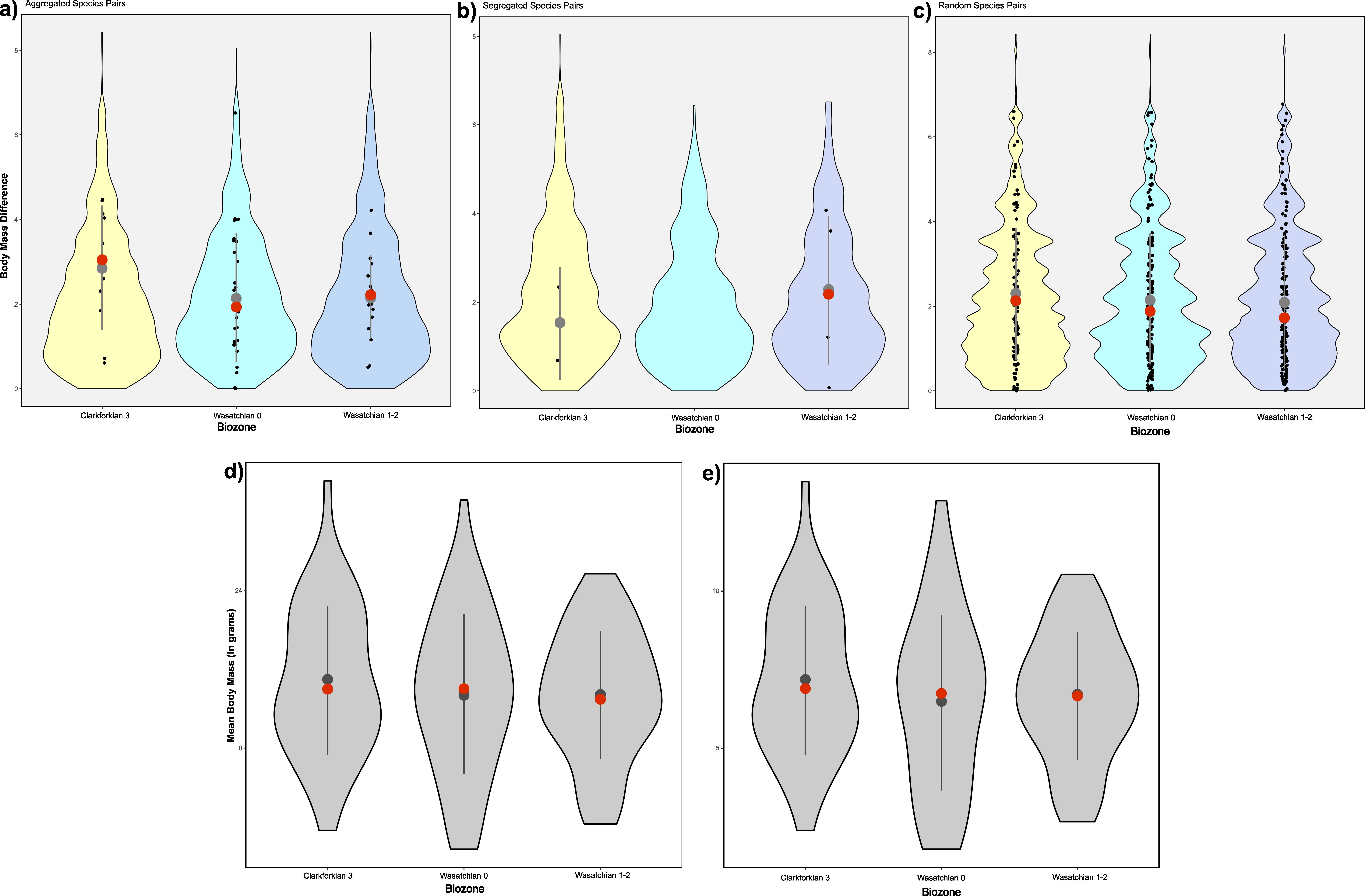

Supplement: Whittingham et al. supplementary material [file S2755095824000251sup001.zip › S2755095824000251sup001/fig5.jpg]
